# Gut Microbes in Immunoglobulin A Nephropathy and Their Potential Therapeutic Applications

**DOI:** 10.3389/fmed.2022.823267

**Published:** 2022-05-17

**Authors:** Yi Wang, Lingling Tian, Lin Sun, Wenjing Zhou, Wenqiang Zhi, Jianbo Qing, Yasin Abdi Saed, Lina Dong, Xiadong Zhang, Yafeng Li

**Affiliations:** ^1^The Third Clinical College, Shanxi University of Chinese Medicine, Taiyuan, China; ^2^College of Traditional Chinese Medicine and Food Engineering, Shanxi University of Chinese Medicine, Jinzhong, China; ^3^School of Medical Sciences, Shanxi University of Chinese Medicine, Jinzhong, China; ^4^The Fifth Clinical Medical College of Shanxi Medical University, Taiyuan, China; ^5^Department of Nephrology, Shanxi Provincial People's Hospital (Fifth Hospital) of Shanxi Medical University, Taiyuan, China; ^6^Core Laboratory, Shanxi Provincial People's Hospital (Fifth Hospital) of Shanxi Medical University, Taiyuan, China; ^7^Shanxi Provincial Key Laboratory of Kidney Disease, Taiyuan, China; ^8^Academy of Microbial Ecology, Shanxi Medical University, Taiyuan, China

**Keywords:** gut microbiota, IgA nephropathy, gut-kidney axis, pathogenesis, therapeutic applications

## Abstract

Microbial ecosystem consists of a complex community of bacterial interactions and its host microenvironment (tissue, cell, metabolite). Because the interaction between gut microbiota and host involves many diseases and seriously affects human health, the study of the interaction mechanism between gut microbiota and host has attracted great attention. The gut microbiome is made up of 100 trillion bacteria that have both beneficial and adverse effects on human health. The development of IgA Nephropathy results in changes in the intestinal microbial ecosystem that affect host physiology and health. Similarly, changes in intestinal microbiota also affect the development of IgA Nephropathy. Thus, the gut microbiome represents a novel therapeutic target for improving the outcome of IgA Nephropathy, including hematuria symptoms and disease progression. In this review, we summarize the effect of intestinal microbiota on IgA Nephropathy in recent years and it has been clarified that the intestinal microbiota has a great influence on the pathogenesis and treatment of IgA Nephropathy.

## Introduction

Microbes are distributed on the surface of the human body and in all body cavities, and they coexist and interact with each other. Intestinal microbes are considered as endocrine organs of the human body. The intestinal microbiota distributed in the human body weighs about 1.5–2.0 kg, among which the number of bacteria, archaea, phages, eukaryotic viruses and fungi is about 100 trillion. It is reported that the number and composition of bacteria in different parts of the gastrointestinal tract is different. The number and types of bacteria in stomach and upper small intestine are small, and the number of bacteria from jejunum to colon gradually increases (1). Among these different bacterial species, the dominant phyla are *Bacteroidetes* and *Firmicutes*, which account for more than 90% of the total microbiome. However, *Actinobacteria, Proteobacteria, Verrucomicrobiota, Clostridium*, and *Cyanobacteria* have low abundance in the gastrointestinal tract of adults, accounting for <10% of the total microbiota (2).

Intestinal microbiota plays an important role in regulating host immunity, food digestion, intestinal endocrine function, drug action, metabolism and elimination of toxins, and can produce a variety of compounds that affect the host (3, 4). As a complex microecosystem, intestinal microbes can participate in the regulation of immunity and pathogen defense, and regulate the metabolism of carbohydrates and lipids in the body, and it is also involved in the metabolism of amino acids, vitamins, which has a significant impact on the physiological reaction of the host (5). The deep reason is that the gut microbial genome is rich in genes involved in the metabolism of carbohydrates, amino acids, methane, vitamins and short-chain fatty acids, many of which is not possessed by the human body itself. When the external environment changes and the intestinal microecological balance is destroyed, it will lead to a variety of host diseases. Changes within the ecosystem of gut microbiome are due to the development of kidney disease, covering CKD, IgA nephropathy, nephrolithiasis, hypertension, acute kidney injury, hemodialysis and peritoneal dialysis (6). Patients with kidney disease are often accompanied by intestinal microflora disorder. On the other hand, intestinal microbiota disorders will further accelerate the process of kidney disease (5–7). This review focuses on the pathogenic association between gut microbiota and IgA nephropathy.

Immunoglobulin A nephropathy (IgAN) is a primary immune glomerulonephritis characterized by deposition of pathogenetic polymeric IgA1 immune complexes in the glomerular mesangium, proliferation of mesangial cells, increased synthesis of extracellular matrix and variable infiltration of macrophages, monocytes and T cells. IgAN mesangial deposition is caused by changes in immunoglobulin A (IgA), which is prevalent in mucosal secretions, so mucosal immunity plays an important role in glomerular diseases (8). It is estimated that 30–40% of IgAN patients will develop end stage renal disease (ESRD) within 20–30 years of diagnosis (9). Some scholars have suggested that the interaction of genetic, epigenetic and environmental factors may lead to the development of IgAN, resulting in the formation of a unique histological biomarker for IgAN, namely glomerular IgA deposition, mainly polymer IgA1, which is more common than other immunoglobulins (10). Many scholars believe that IgAN is associated with abnormal immune response, bacterial infection and inflammation, but the detailed pathogenesis of IgAN remains to be further studied and explored. In recent years, multiple omics studies have found that mucosal microbial immunity is involved in the pathogenesis of IgAN, among which respiratory tract and digestive tract infection can cause or aggravate IgAN (11), and intestinal microbiota is likely to be the most important environmental factor in the pathogenesis of IgAN. However, there is still a lack of systematic academic results to clarify the process of intestinal microbiome imbalance leading to IgAN. Therefore, in this review, we discussed changes in intestinal microbiome composition in IgA nephropathy and the role of microorganisms and their metabolites in IgA nephropathy, and further explored the association between intestinal microbiome and IgA nephropathy.

## Role of Gut-Kidney Axis in IgA Nephropathy

A vast array of complex and abundant microorganisms live in the human intestine, collectively referred to as the intestinal microbiome (3). The flora consists of 10^14^ bacteria with a biomass of 2 kg. The researchers (12) presented a comprehensive collection of 7,758 gut bacterial isolates paired with 3,632 genome sequences and longitudinal multi-omics data. Gut microbiota has physiological functions related to nutrition, immune system and host defense. Previous studies have reported significant changes in the gut microbiome of IgAN patients compared to healthy controls, results from the report have associated the gut microbiota with the development of IgAN although the mechanisms of how they function remain elusive. Zhong et al. (13) analyzed the fecal intestinal microbiota of IgAN patients in the Chinese population. Cladogram and LEfSe analyses suggested that the relative abundances of the dominant bacterial communities were different. *Subdoligranum* was the most abundant genus in healthy control patients (HCs), and *Bacteroides* was dominant genus in IgAN patients. They (13) also found a decrease in beneficial bacteria such as *Lachnoclostridium, Blautia, Prevotella 9, genus_Eubacterium hallii* and *Bifidobacterium* decreased levels of these beneficial bacteria may compromise the immune regulatory function in the intestines. More and more studies indicate that the gut-kidney axis may be involved in the pathogenesis of many diseases. There are some studies (13–16) have reported intestinal microbiome dysregulation in IgAN patients. Additionally, changes in the composition of the intestinal microbiota and increased IgA highly coated bacteria were indicative of potential involvement of intestinal dysbiosis to the systemic IgA level and, consequently, the IgAN progression. 16S rRNA sequencing revealed differences in microbiome composition from phylum to genus level between IgAN patients and blank control patients (17). Germ-free mice (GF) have a reduced number of gut associated lymphoid tissues (GALT), and it reduced the host's immunity accordingly (18). Studies in (GF) showed that the gut microbiota is required for normal immune system maturation, including GALT development (19). Microorganisms on the mucosal surface are in close contact with the intestinal epithelium and have a great influence on the regulation of GALT by affecting the intestinal barrier to pathogens and the host immune system (19).

Studies have shown that microorganisms and their metabolites play a key role in maintaining the immune balance of GALT. Microbiota affects the function of intestinal mucosa and the host's systemic immune response. Recent studies have shown that GALT plays an important role in the development of IgAN. Intestinal microbiota plays a key role in the development of IgAN in B cell activation factor of the TNF family (BAFF) overexpressing transgenic mice (20). It is suggested that genetic and microbial factors interact with each other to induce functional changes of intestinal mucosal immune system, thus promoting the development of IgAN (8).

## Effects of IgAN on the Gut Microbiome

Internal factors, such as changes in physiological responses to disease processes, will lead to changes in the composition and function of microbial communities, thus affecting changes in intestinal microecosystems (21).

Some scientists have concluded that significant changes in the composition of intestinal microbiota in IgAN patients nephropathy has changed significantly. Jørgensen et al. (22) and Fransen et al. (23) considered that bacterial biodiversity changes with changes in the relative abundance of a particular bacterial group, genus, or species as well as genetically and environmentally driven and depends on the amount and diversity of innate IgAs present at birth. According to the information available so far as well and the estimation of sparsity and Chao1 diversity index, the microbial diversity of IgAN patients is lower than that of normal people (17). Changes in bacterial biodiversity could indirectly reflect the health of the host intestinal ecosystem (24).

Two sequencing technology, 16S rDNA sequencing and metagenomic sequencing, have commonly been used for identification of microbial communities compositions and their relative abundance, and related deep, longitudinal shotgun sequencing has enabled large-scale characterization of personalized microbiome. In this section, we list the correlation between intestinal microbiome at phylum and genus levels and IgA nephropathy (Table 1).

**Table 1:** Altered gut microbial compositions associated with IgAN-16S sequencing.

**Species**	**Illness-associated changes in gut microbial abundance**		**References**
	**Increase**	**Decrease**	
**Phylum abundance**			
Homo sapiens	*Proteobacteria, Fusobacteria* and *Actinobacteria*	*Firmicutes, Bacteroidetes, Euryarchaoeota* and *Ruminococcaceae*	(25, 26)
Homo sapiens	*Proteobacteria, Fusobacteria, Bacteroidetes* and *Lachnoclostridium*	*Bifidobacterium* and *Enterococcus faecalis*	(13)
Homo sapiens	*Fusobacteria* and *Proteobacteria*	*Synergistetes*	(27)
Homo sapiens	*Proteobacteria* and *Candidate_division_TM7*	*Synergistetes*	(28)
**Genus abundance**			
Homo sapiens	*Cyanobacteria, Euryarchaeota, Betaproteobacteriales, Sutterellaceae, Methanobacteriales, Lachnospiraceae, Ruminococcaceae, Eubacteriaceae* and *Enterobacteriaceae*	*Bifidobacterium, Clostridium, Enterococcus* and *Lactobacillus*	(25)
Homo sapiens	*Escherichia-Shigella, Bacteroides, Fusobacteria, Streptococcus* and *Enterococcus*	*Bifidobacterium, Faecalibacterium, Blautia, Subdoligranulum Prevotella 9* and *Eubacterium hallii*	(29)
Homo sapiens	*Escherichia-Shigella* and *Defluviitaleaceae_incertae_sedis*	*Synergistetes, Lachnospiraceae Roseburia, Haemophilus*, and *Clostridium_sensu_stricto_1*	(28)
Homo sapiens	*EscherichiaShigella, Bacteroides, Fusobacteria, Enterobacteriaceae, Hungatella* and *Eggerthella*	*Synergistetes* and *Lachnospiraceae*	(27)
Homo sapiens	*Escherichia-Shigella* and *Bacteroides*	*Bifidobacterium, Faecalibacterium, Eubacterium hallii* and *Blautia*	(13)
Homo sapiens	*Ruminococcaceae, Lachnospiraceae, Eubacteriaceae, Streptococcaeae, Sutterellaceae* and *Enterobacteriaceae*	*Bifidobacterium* and *Lactobacillus*	(25)

### Variation in Phylum-Level Abundance

Zhong et al. found that the relative abundance of *Bacteroidetes, Lachnoclostridium* and *Fusobacteria* were also high in IgAN patients (13). Some researchers found that *Proteobacteria* were increased in IgAN patients (13, 25–28). In addition, De Angelis and Sugurmar et al. found that compared with the healthy control group, the number of *Actinobacteria* in patients in IgAN group was significantly increased (25, 26). *Fusobacteria* phylum was significantly increased (25–27). Dong et al. found that *Candidate_division_TM7* were overrepresented in IgAN patients (28).

Eckburg et al. and Sugurmar et al. found that *Firmicutes* and *Bacteroidetes* are the main phyla of healthy human intestinal microbiota, which showed a significant downward trend in IgAN patients (26, 30). In addition, *Euryarchaoeota* phylum was reduced in the IgAN group (26). Hu et al. and Dong et al. found that compared to the healthy controls, *Synergistetes* were significantly underrepresented in patients with IgAN (27, 28).

### Variation in Genus-Level Abundance

Several scientists concluded that a higher proportion of the genera *Escherichia-shigella* had significantly higher levels in IgAN patients than in HCs patients (27–29). Hu et al. and Wu et al. found that *Bacteroides* were increased in IgAN (27, 29). Hu et al. also found in their study that the abundance of *Enterobacteriaceae, Hungatella*, and *Eggerthella* increased in IgAN patients, all of which possess pathogenic potential (27). Dong et al. observed that *Defluviitaleaceae_incertae_sedis* were enriched in IgAN (28). According to Wu et al., *Streptococcus* and *Enterococcus* have a low proportion of microorganisms in HCs, but they are very abundant in feces samples of IgAN patients, indicating that these two microorganisms may be significant characteristics of IgAN (29). In addition, De Angelis et al. found that the percentage of some genera/species in *Ruminococcaceae, Lachnospiraceae, Eubacteriaceae* and *Streptococcaeae* was higher in IgAN (25).

Several studies concluded that *Bifidobacterium* and *Faecalibacterium* showed a decreasing trend in IgAN group (13, 29). Moreover, Zhong et al. found that *Blautia, Subdoligranulum, Prevotella 9, genus_Eubacterium Hallii, Enterococcus faecalis* and *Lachno-clostridium* were also significantly reduced (13). Hu et al. and Dong et al. found that the fecal microbiome richness of IgAN patients was significantly lower than that of healthy controls. Compared with healthy controls, the abundance of *Synergistetes* and *Lachnospiraceae* were reduced (27, 28). In addition, Hu et al. also found the abundances of the genus *rectale_group, generarectale_group, Barnesiella, Ruminococcaceae_NK4A214_group, Prevotellaceae_NK3B31_group, Prevotellaceae_UCG-001 Coprococcus_2* and *Pyramidobacter* were decreased in the IgAN group than in the healthy group (27). At the genus level, Dong et al. observed that four genera, namely, *Roseburia, Clostridium_sensu_stricto_1, Haemophilus*, and *Fusobacterium* were deceased in IgAN (28).

Interestingly, among stool samples from non-progressor (NP) and progressor (P) patients, Sutterellaceae and Enterobacteriaceae species were nearly the highest (25). Authors also found that Bifidobacterium, Clostridium, Lactobacillus species and other probiotics were significantly reduced in the fecal samples of IgAN non-progressive and/or progressive patients (25).

At present, the causal relationship between the changes in the gut flora and IgAN cannot be confirmed. However, these results mentioned above suggest that the development of IgAN may cause changes in the composition and community richness of the intestinal flora. The changes in microbes may induce chronic infection and inflammatory responses, leading to IgAN, and the imbalance of intestinal flora may be an important factor in the severity of IgAN. The gut microbiota is likely to be a potential trigger for IgAN, suggesting that certain specific gut microbiota may be a potential therapeutic target for IgAN. Thus, this finding determined the differentially distributed microbial communities as biomarker candidates for IgAN. However, further research is needed to use the microbiota as a biomarker to observation the development of IgAN and specific strains needed to induce or treat IgAN.

## Roles of Metabolites from Microbiota in IgAN

Human indigestible plant polysaccharides are the main substrate for microbial growth in the colon, while butyrate and other microbial fermentation products are important energy sources for the host (31, 32). The gut microbiome promotes host physiology by producing numerous metabolites. These metabolites act in the host as signaling molecules and substrates for metabolic reactions (33). Anaerobic bacteria activate their machinery under specific intestinal conditions, consisting of key enzymes and metabolic pathways that metabolize complex carbohydrates, resulting in the production of metabolites such as SCFA.

### Short Chain Fatty Acids

Microbial metabolites, including SCFA and butyrate, have attracted considerable attention in IgAN's research. SCFA play a number of important roles in maintaining health, such as serving as a special nutrient and energy source for intestinal epithelial cells, protecting the intestinal mucosal barrier, reducing inflammation, and enhancing the motor capacity of the gastrointestinal tract (34). SCFA include acetate, propionate, and butyrate. SCFA is absorbed by the host and used as energy, and butyrate is the main energy source of intestinal epithelial cells (35).

Studies on germ-free mice and GPR43-deficient (Gpr432/2) mice have indicated SCFAs binding of GPR43 potentially provides a molecular link between gastrointestinal bacterial metabolism, and immune and inflammatory responses and, can affect various physiological processes, leading to changes in microbial community structure and function (36, 37). SCFAs were reduced in patients with IgAN, including Group *NK4A214 of Lactococcus* (*Ruminococca-ceae*), group 2 of fecal coccus (*Coprococcus*) and group *FCS020 of Lactococcus* (*Lachnospira-ceae*) (27). The levels of acetic acid, propionic acid, butyric acid, isobutyric acid and caproic acid in IgAN patients were significantly lower than those in the control group. Butyric acid and isobutyric acid are negatively correlated with uric acid. Butyric acid was negatively correlated with urea nitrogen (38). There was a negative correlation between caproic acid and 24 h urinary protein level. SCFAs levels in feces of IgAN patients were decreased and correlated with clinical parameters and intestinal microbiota (39).

### Polyunsaturated Fatty Acid

Polyunsaturated fatty acids (PUFAs) are a group of critical nutrients that modulate brain development and cognition as well as regulate inflammatory and immune responses (40). Wu et al. (29) detected a decrease in polyunsaturated fatty acids in IgAN patients, which may cause inflammation, oxidative stress, and tubulointerstitial fibrosis in the remnant kidney. The changes in gut microbiota also affected metabolism and the absorbance of PUFA, resulting in a unique metabolic system in IgAN patients. Since fish oil is a source of PUFA, consumption of the long-chain n-3 PUFA from fish oil could suppresses the trichothecene mycotoxin deoxynivalenol (DON)-induced IgAN in mice, which concurs with the proposed anti-inflammatory action of these fatty acids. These results demonstrating that PUFA consumption retards the renal function loss in IgAN Patients (41). Some studies (42, 43) found that combined treatment with renin–angiotensin system blockers and polyunsaturated fatty acids in proteinuric IgA nephropathies contribute to percent reduction of proteinuria from the baseline and associated with changes in glomerular filtration rate (GFR), blood pressure, serum triglycerides and erythrocyturia. In addition, Zivkovic et al. speculated that n-3 PUFA supplementation is associated with improved kidney function in IgAN (44). Increased levels of pro-inflammatory arachidonic acid metabolites, decreased levels of anti-inflammatory unsaturated fatty acids and fatty acid derivatives may play a role in persistent intestinal inflammatory and immune activation in IgAN patients, which might cause immune hyperactivation in IgAN patients (45).

### Other Metabolites

Serum metabolic profiling using PCA score plots and OPLS-DA model analysis confirmed that the two groups were clearly separated, which were employed to seek out critical markers in the IgAN model. Six key metabolites have been identified in stool and serum samples. These include metabolites—bilirubin, trimethoprim, stearamide, phenylalanine, cis-9, 10-epoxystearic acid and phosphatidylethanolamine (PE Lyso 17:0) (29). These biomarkers of metabolites were mainly involved in lipid metabolism, signal transduction, carbohydrate metabolism, amino acid metabolism, and biosynthesis of other secondary metabolites (29). They may be a core metabolite of IgAN and may be an important mediator between intestinal and blood circulation (46).

Changes in IgAN intestinal microbiota affect the metabolism and absorption of microbiota related metabolites. The metabolic pathways enriched by the metabolites were consistent with the potential metabolic function predicted by the intestinal flora (29). Therefore, intestinal flora could affect the development of IgAN by altering metabolites.

## Microbiota-Targeted Therapeutics

Increasing evidence indicate that gut microbiota plays a very significant role in the progression of IgAN. Thus, the gut microbiota has become an ideal target for the prevention and treatment of diseases. Therapeutic measure designed to manipulate gut microbiota composition and/or their metabolisms have been developed, including antibiotics, probiotics and fecal microbiota transplantation (FMT) as well as Chinese herbal medicine (32). To some extent, these strategies described above have significantly improved hematuria and proteinuria symptoms in some IgAN patients in both theoretical and clinical applications.

### Antibiotics and Drug Therapy

The microbiome can be regulated by antibiotics and has been used to alter the microbiome. Chemouny JM showed that broad-spectrum antibiotics reduced human immunoglobulin A1 (hIgA1)—mouse immunoglobulin G (mIgG) complex serum levels in the circulating system and proteinuria levels, and prevented IgA1 mesangial deposition and glomerular inflammation without affecting serum IgA levels in humanized α1^Ki^-CD89^TG^ mice. This suggests that nephrogenic IgA is regulated by intestinal microbiota (47). Microbiota plays an important role in mucosa-associated lymphoid tissue (MALT) regulation. Rifaximin is a non-absorbable oral antibiotic that induces positive regulation of the gut microbiome and facilitates the growth of host-beneficial bacteria. Rifaximin decreased urinary protein-creatinine ratio, serum hIgA1-SCD89 and mIgG-hIgA1 complex levels, and hIgA1 glomerular deposition in IgAN humanized α1^Ki^-CD89^TG^ mouse model, suggesting that Rifaximin may play a role in the treatment of this disease (48). Pan ZY study showed that short-term high-dose hydroxychloroquine (HCQ) stimulation significantly changed the structure, richness and community diversity of intestinal microbiota in mice, increasing the relative abundance of Bacteroides and decreasing the relative abundance of Firmicutes (49).

### Probiotics and Prebiotics Treatment

A large number of studies have been carried out to add probiotics or prebiotics to the diet as a means of regulating the intestinal microbiota and the host immune response (50), which may be a useful tool for repairing some microbial gaps, such as *Lactobacillus* and *Bifidobacteria* (26). Huang et al., Flint et al., showed that some *Lactobacillus* and *Bifidobacterium* also produce lactic acid and acetic acid to reduce IgA production (51, 52). Soylu et al. showed that A. brassica could prevent oral poliovirus vaccine-induced IgA nephropathy in mice, suggesting that A. brassica might down-regulate the response of systemic IgA to intestinal antigen stimulation (53).

Consumption of probiotics is a widely accepted method of influencing the microbiome and regulating its health or disease function. Aindelis G study to identify the effects of probiotics *Lactobacillus casei ATCC393* on intestinal microbiota and microbial origin in mice. Next generation sequencing analysis showed that the use of *Lactobacillus casei* ATCC393 changed the composition and population of gut microbes. In lactobacilli-fed animals, abundance of taxa classifed as *Muribaculaceae* was increased, while abundance of taxa classified as *Lachnospiraceae* and *Oscillospiraceae* was decreased. In addition, IgA production and lactic acid concentration were significantly increased, while acetic acid level was significantly decreased. IgA is the most common antibody type in the intestinal mucosa. The increase of IgA is immune to the gut and better protected against infection, but excessive IgA secretion may lead to the occurrence or aggravation of IgAN. Results showed that *Lactobacillus casei ATCC393* can act as a potential strain to regulate intestinal homeostasis and can also perform dietary interventions to improve health status (54).

### Fecal Microbiota Transplantation

Fecal microbiome transplantation (FMT) is the transfer of donor fecal samples into the gastrointestinal tract of patients whose microbiota has been depleted to restore their normal gastrointestinal microbiota. Studies (55, 56) have reported that patients with IgA nephropathy effectively relieved their clinical symptoms after FMT treatment, with 24-h urinary protein reduced to less than half of baseline, serum albumin increased, and renal function stabilized. FMT changes the intestinal microbiota of IgAN patients, with *Proteobacteria* decreasing and *Prevotella* increasing during and after FMT (55). The serum BAFF level of FMT mice in IgAN patients was higher. There were significant differences in the composition of major phyla and volatile organic compounds in the intestinal microbiota of mice. Changes in FMT microbiota affect IgAN phenotype, which may be a new pathway for treatment of IgA nephropathy (56).

### Chinese Herbal Medicine

Chinese herbal medicine has been widely used in the diagnosis and treatment of Traditional Chinese Medicine (TCM). From ancient times to modern times, Chinese herbal medicine is used in prevention and treatment of diseases (57) and is a plant-derived material or preparation that can treat or benefit human health. There is increasing evidence that the interaction between gut microbiota and herbal components plays a crucial role in herbal therapy. And preliminary studies have found that these herbs could help treat diseases related to the gut microbiome.

Artemisinin (ART) is the major active chemical ingredient from the classical herbal medicine of Artemisia apiacea. Previous studies have shown that ART has anti-malaria, immunomodulatory, anti-tumor, anti-inflammatory and other pharmacological activities (58). The results of Bai's study showed that Artemisinin and Hydroxychloroquine (AH) can effectively improve renal injury in IgAN rats by improving renal dysfunction, reducing 24 h urinary protein, and reducing the deposition level of IgA and IgG immune complexes in mesangium (58). Combination of ART and HCQ has the advantage of synergism and detoxification and AH could exert stronger therapeutic effect than ART or HCQ as a standalone application on the treatment of IgAN (59). Therefore, Artemisinin plays an indispensable role in the treatment of IgAN.

Zhen Wu Tang is a classic herbal medicine prescription that has been clinically used in China for more than 1,000 years. It is used in the treatment of chronic kidney disease (CKD) and can relieve edema, dysuria and oliguria and other symptoms (60, 61). Analysis of fecal and serum samples of IgAN rats by 16S rDNA sequencing and untargeted metabolomics revealed changes in intestinal microbiota composition and metabolites. Feeding of Zhen Wu Tang to IgAN model rats significantly changed the gut microbiota with increased the relative abundance of microbial species at the family level, including *Peptostreptococcaceae* and *Family_XIII* while suppressing the abundances of *Cyanobacteria* and *Proteobacteria*. Moreover, the model group had higher abundance of *Lachnospiraceae, Lactobacillaceae, Erysipelotrichaceae*, and *Bacteroidaceae* relative to the normal group; ZWT intervention reduced these levels (46). Zhen Wu Tang can improve the microecological imbalance and reduce kidney damage in IgAN rats (46).

## Conclusion

**Figure 1:**
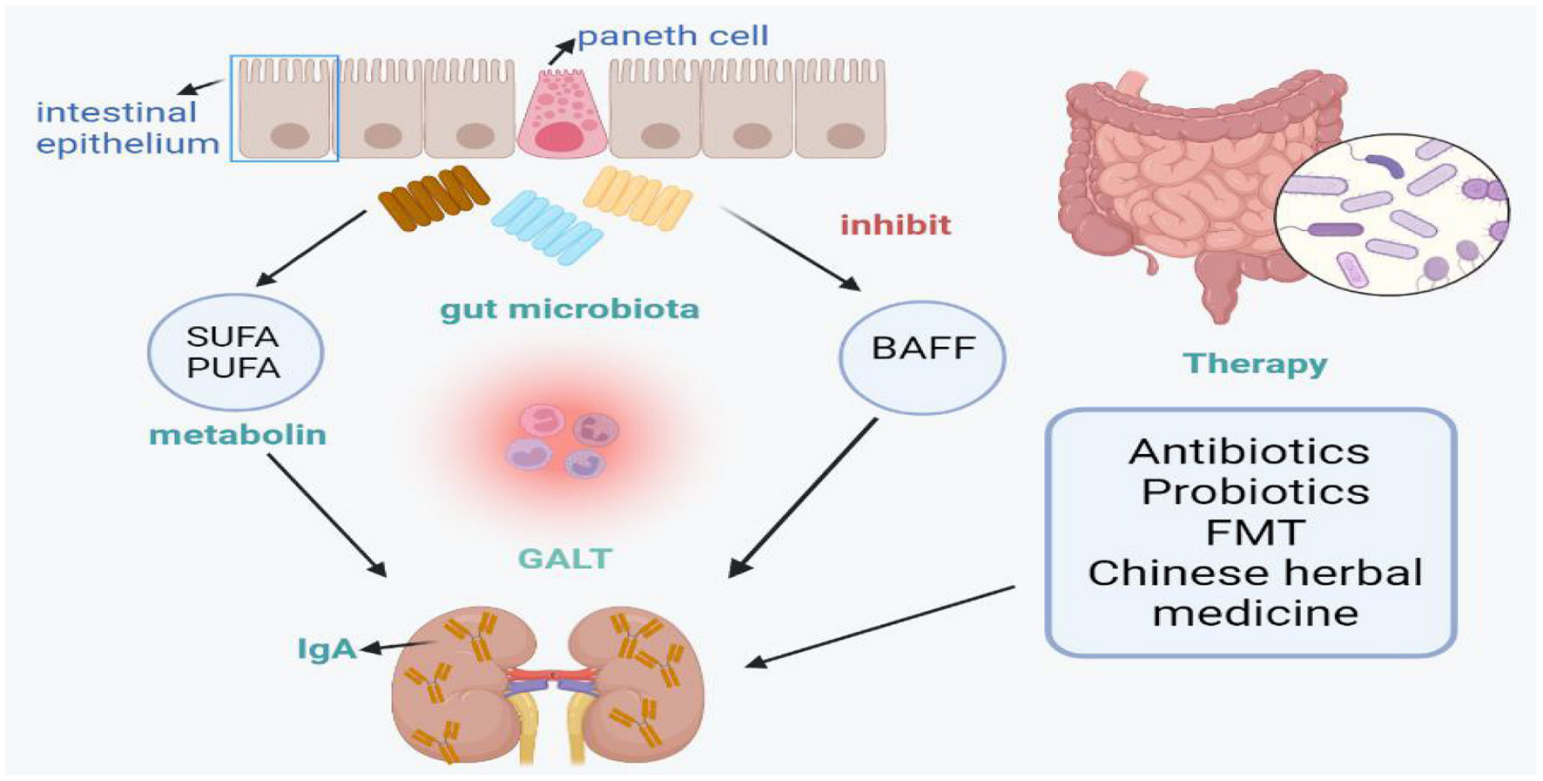
Microbiota and associated metabolites involved into the pathogenesis of IgAN.

This review summarizes the changes in intestinal microbial diversity and phylum in IgA nephropathy, and introduces the prevention and treatment of IgA nephropathy from the perspective of intestinal microbes, including antibiotics, probiotics, fecal transplantation and Chinese herbal therapy, aiming to control the composition and metabolism of intestinal microbiota (Figure 1). The imbalance of intestinal microflora and its metabolites is related to the occurrence and development of IgA nephropathy. In conclusion, we found that *Bacteroides* and *Escherichia-Shigella* levels were significantly higher in patients with IgAN, while *Bifidobacterium* and *Blautia* levels were lower. Accordingly, we considered that we should focus on these bacterial flora in future studies. However, identification of specific microbe strains rather than a general bacterial community is helpful to elucidate the contributions of specific microorganisms to disease progression. Thus, further research is needed to use the microbiota as a biomarker in the development of IgAN and specific strains needed to induce or treat IgAN. The contribution of the microbiome to the IgAN process suggests that targeted therapies may be valuable for the treatment of IgAN, but further studies of the functional role of gut microbiome composition are needed.

## Author Contributions

YW: conceptualization, methodology, resources and writing-original draft preparation. LT: literature search. LS, WZho, WZhi, JQ, YS, and LD: critical feedback and editing the subsequent revisions. XZ: organization and assist in polishing. YL: article management, supervision, writing review and editing. All authors contributed to the article and approved the submitted version.

## Funding

This research was supported by the National Natural Science Foundation of China (Grant No. 82170716) and Research Project Supported by Shanxi Scholarship Council of China.

## Conflict of Interest

The authors declare that the research was conducted in the absence of any commercial or financial relationships that could be construed as a potential conflict of interest.

## Publisher's Note

All claims expressed in this article are solely those of the authors and do not necessarily represent those of their affiliated organizations, or those of the publisher, the editors and the reviewers. Any product that may be evaluated in this article, or claim that may be made by its manufacturer, is not guaranteed or endorsed by the publisher.
